# Supplementary figures and images for: The relation between urinary sodium and potassium excretion and risk of cardiovascular events and mortality in patients with cardiovascular disease
Source: PLoS One. 2022 Mar 17;17(3):e0265429. doi: 10.1371/journal.pone.0265429 (PMC8929575; doi:10.1371/journal.pone.0265429)

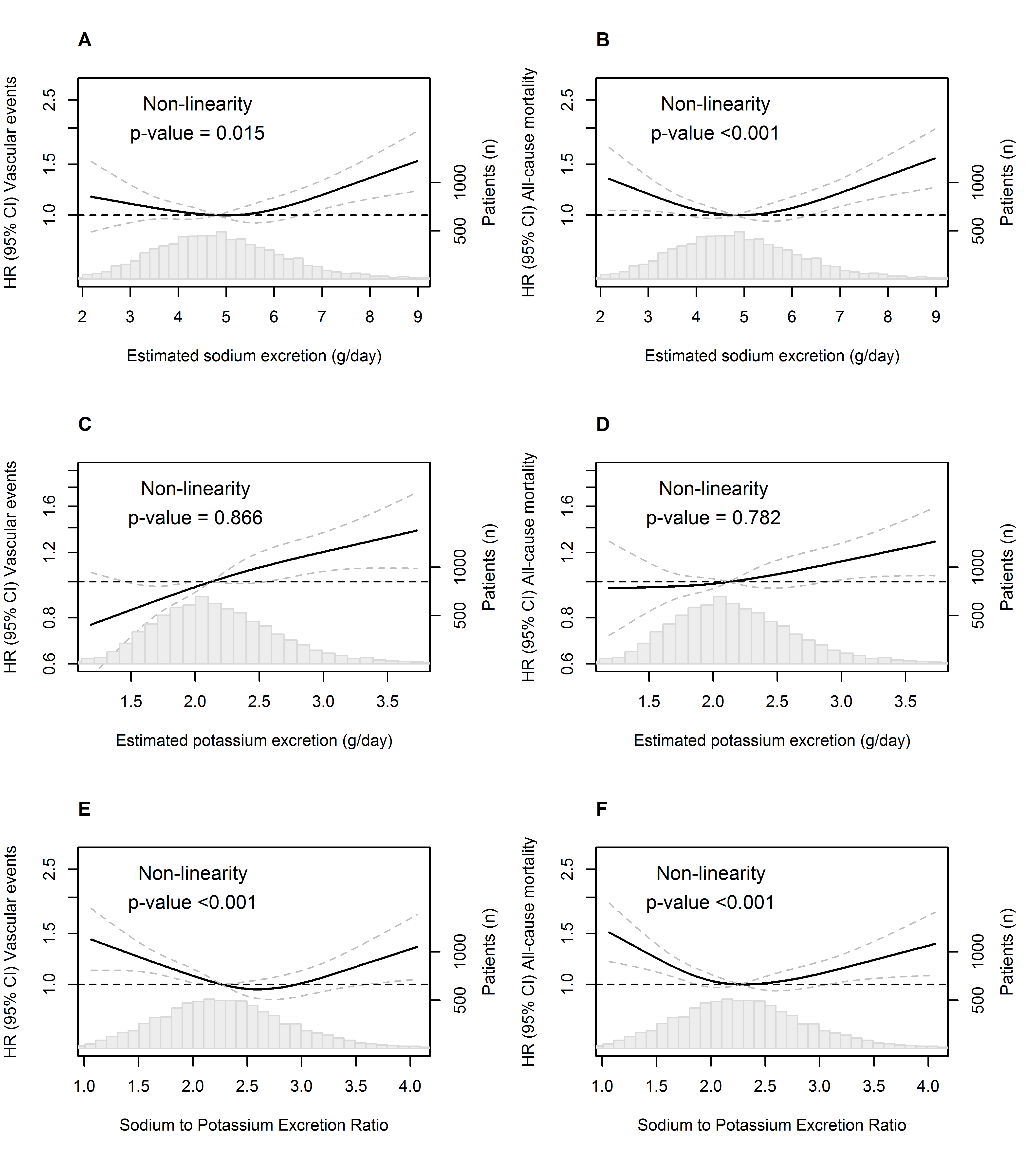

Supplement: S1 Fig — Restricted-cubic-spline plots of association between estimated 24-hour urinary excretion of sodium (A-B), potassium (C-D), and their ratio (E-F) and recurrent MACE (left column) and all-cause mortality (right column). Histograms demonstrate distributions of different salt measures. The median of each salt measure (4.80 g/day, 2.12 g/day and 2.27 for sodium, potassium and their ratio, respectively) was taken as a reference (HR = 1.0). Spline curves were plotted between the 1st and 99th percentile of the corresponding salt measure. Dotted lines represent 95% confidence intervals. All plots were adjusted for age, sex, current smoking, BMI (kg/m2), presence of diabetes, eGFR, and non-high-density lipoprotein cholesterol. HR = Hazard ratio. (PNG) [file pone.0265429.s001.png]

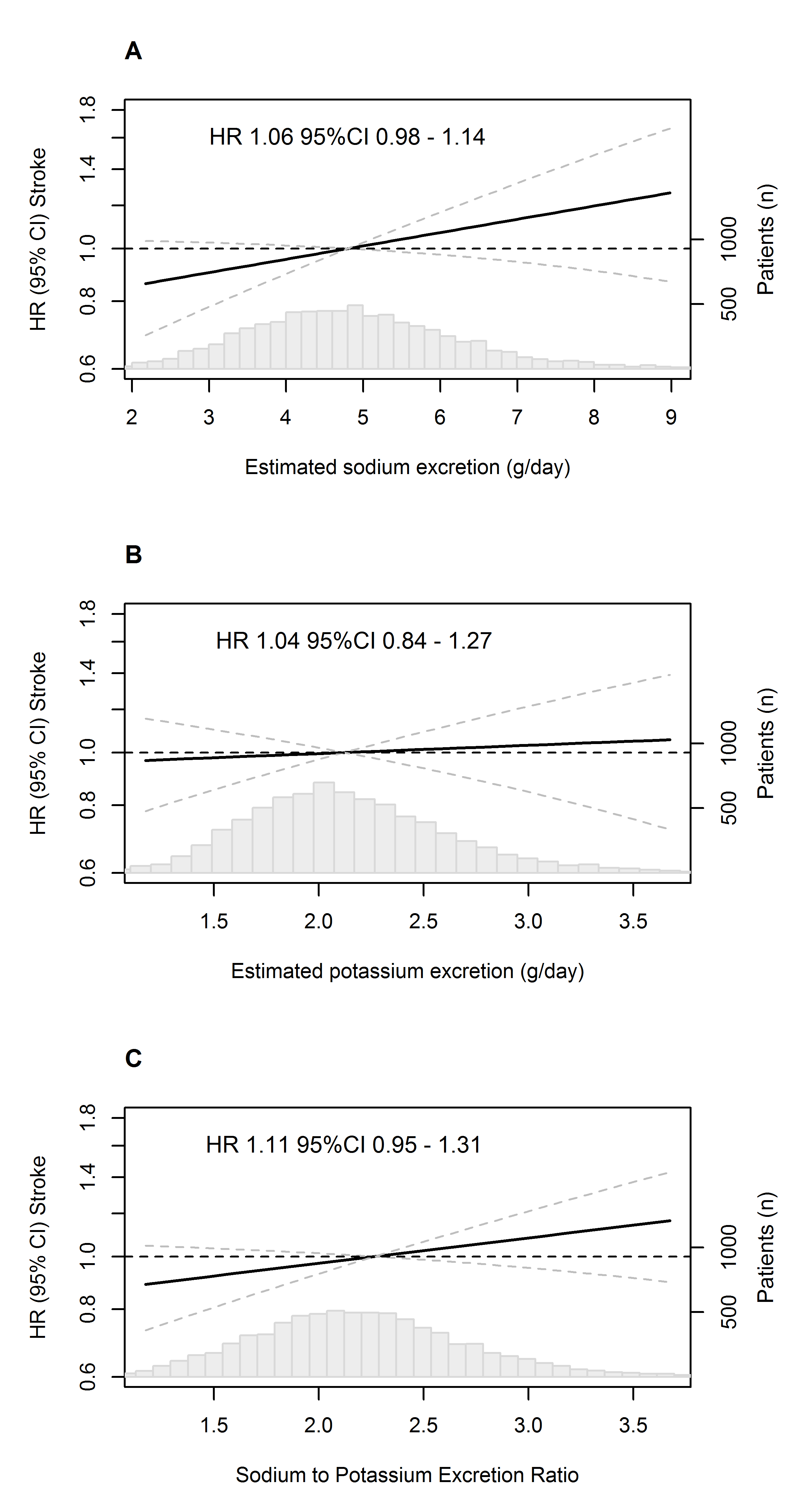

Supplement: S2 Fig — A. Relation between 1 gram/day higher estimated 24-hour urinary sodium excretion and the occurrence of stroke. B. Relation between 1 gram/day higher estimated 24-hour urinary potassium excretion and the occurrence of stroke. C. Relation between 1 unit higher sodium-to-potassium excretion ratio and the occurrence of stroke. Histograms demonstrate distributions of different salt measures. The median of each salt measure (4.80 g/day, 2.12 g/day and 2.27 for sodium, potassium and their ratio, respectively) was taken as a reference (HR = 1.0). All hazard ratios were plotted between the 1st and 99th percentile of the corresponding salt measure. Dotted lines represent 95% confidence intervals. All plots were adjusted for age, sex, current smoking, BMI (kg/m2), presence of diabetes, eGFR, and non-high-density lipoprotein cholesterol. HR = Hazard ratio. (PNG) [file pone.0265429.s002.png]

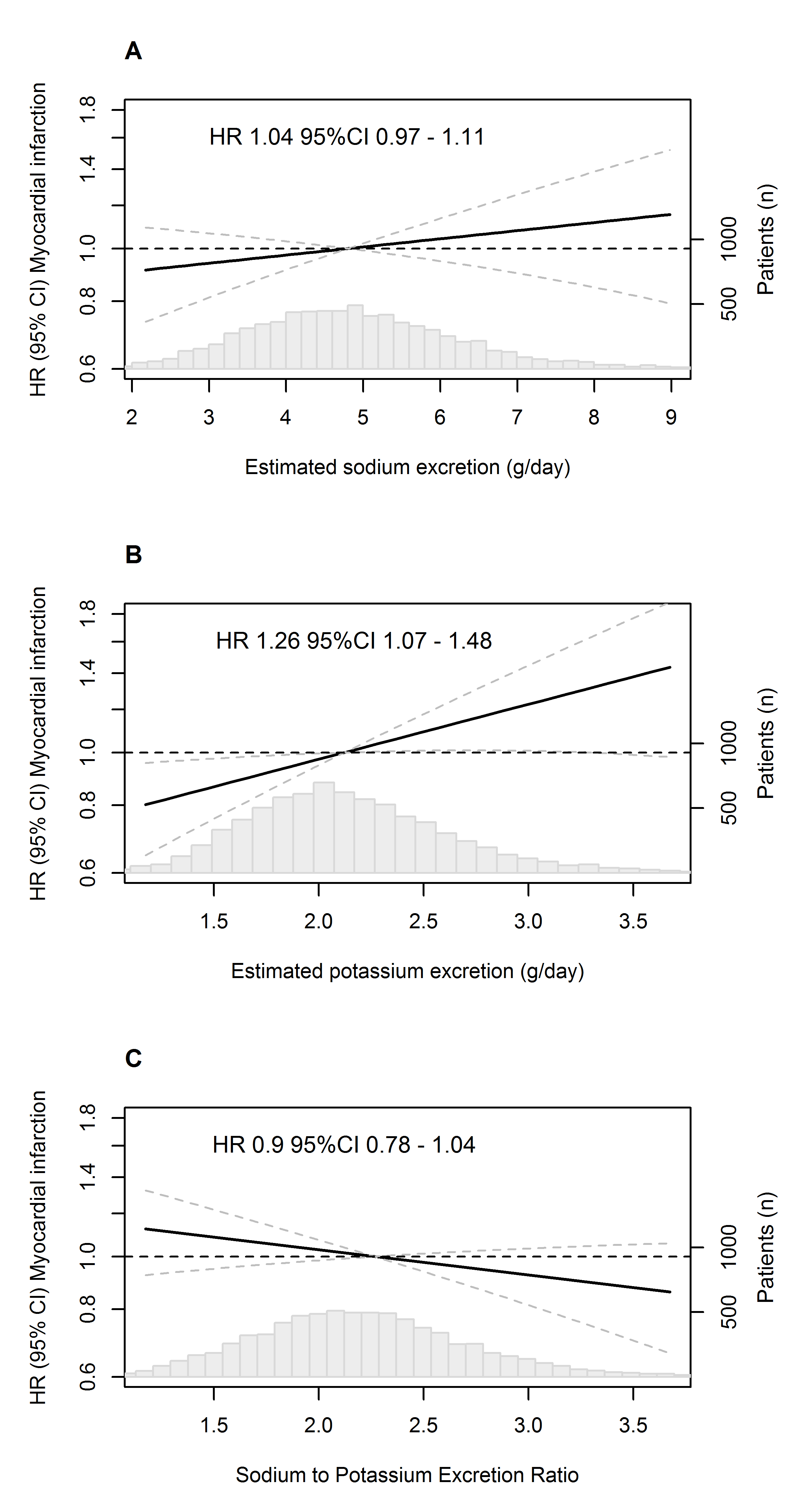

Supplement: S3 Fig — A. Relation between 1 gram/day higher estimated 24-hour urinary sodium excretion and the occurrence of myocardial infarction. B. Relation between 1 gram/day higher estimated 24-hour urinary potassium excretion and the occurrence of myocardial infarction. C. Relation between 1 unit higher sodium-to-potassium excretion ratio and the occurrence of myocardial infarction. Histograms demonstrate distributions of different salt measures. The median of each salt measure (4.80 g/day, 2.12 g/day and 2.27 for sodium, potassium and their ratio, respectively) was taken as a reference (HR = 1.0). All hazard ratios were plotted between the 1st and 99th percentile of the corresponding salt measure. Dotted lines represent 95% confidence intervals. All plots were adjusted for age, sex, current smoking, BMI (kg/m2), presence of diabetes, eGFR, and non-high-density lipoprotein cholesterol. HR = Hazard ratio. (PNG) [file pone.0265429.s003.png]

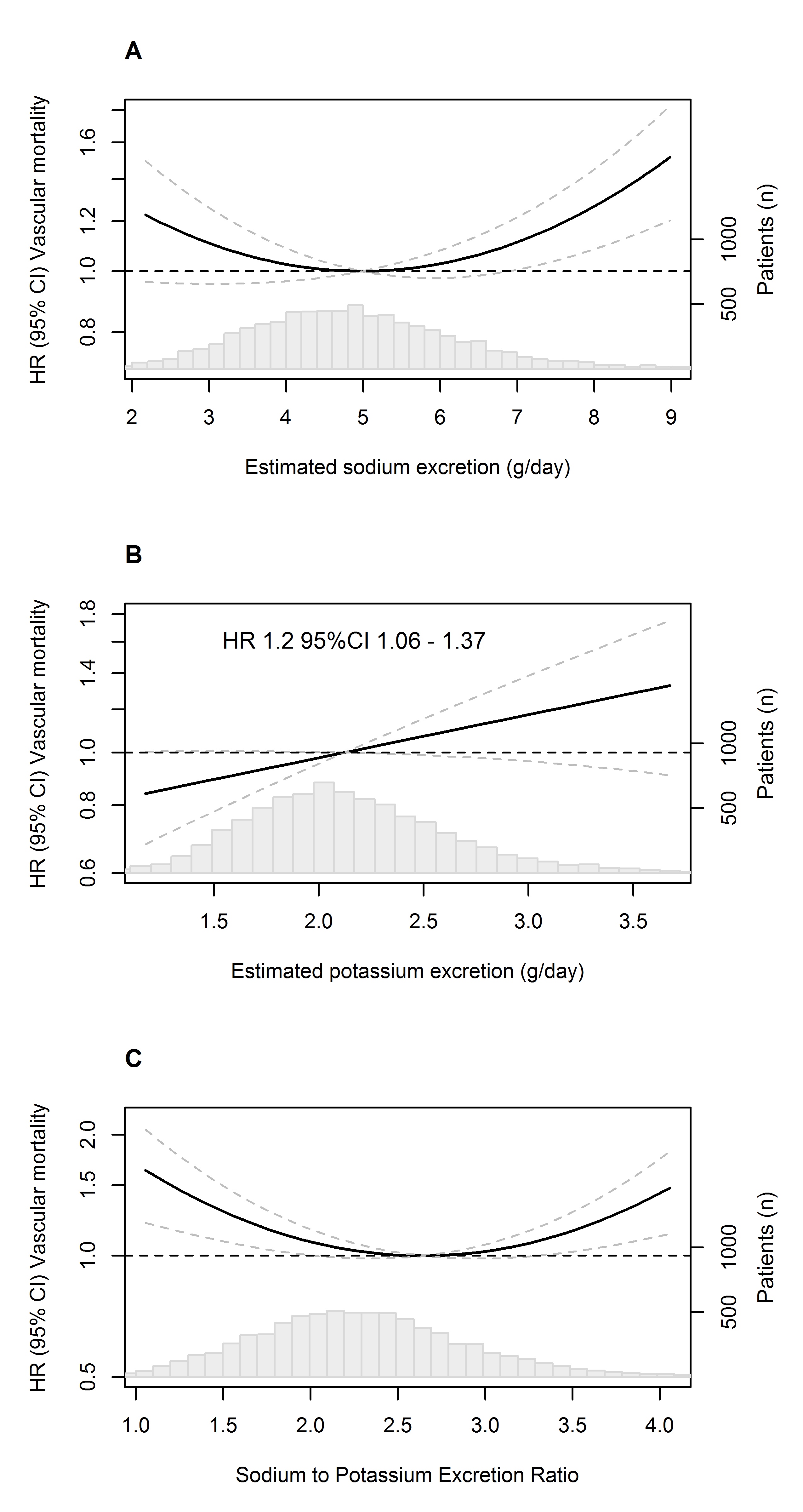

Supplement: S4 Fig — A. Relation between estimated 24-hour urinary sodium excretion and vascular mortality (linear term P<0.01; non-linear term P<0.01). Nadir: 4.98 g/day. B. Relation between 1 gram/day higher estimated 24-hour urinary potassium excretion and vascular mortality. C. Relation between sodium-to-potassium excretion ratio and vascular mortality (linear term P<0.01, non-linear term P<0.01). Nadir 2.64. Histograms demonstrate distributions of different salt measures. All hazard ratios were plotted between the 1st and 99th percentile of the corresponding salt measure. Dotted lines represent 95% confidence intervals. All plots were adjusted for age, sex, current smoking, BMI (kg/m2), presence of diabetes, eGFR, and non-high-density lipoprotein cholesterol. HR = Hazard ratio. (PNG) [file pone.0265429.s004.png]

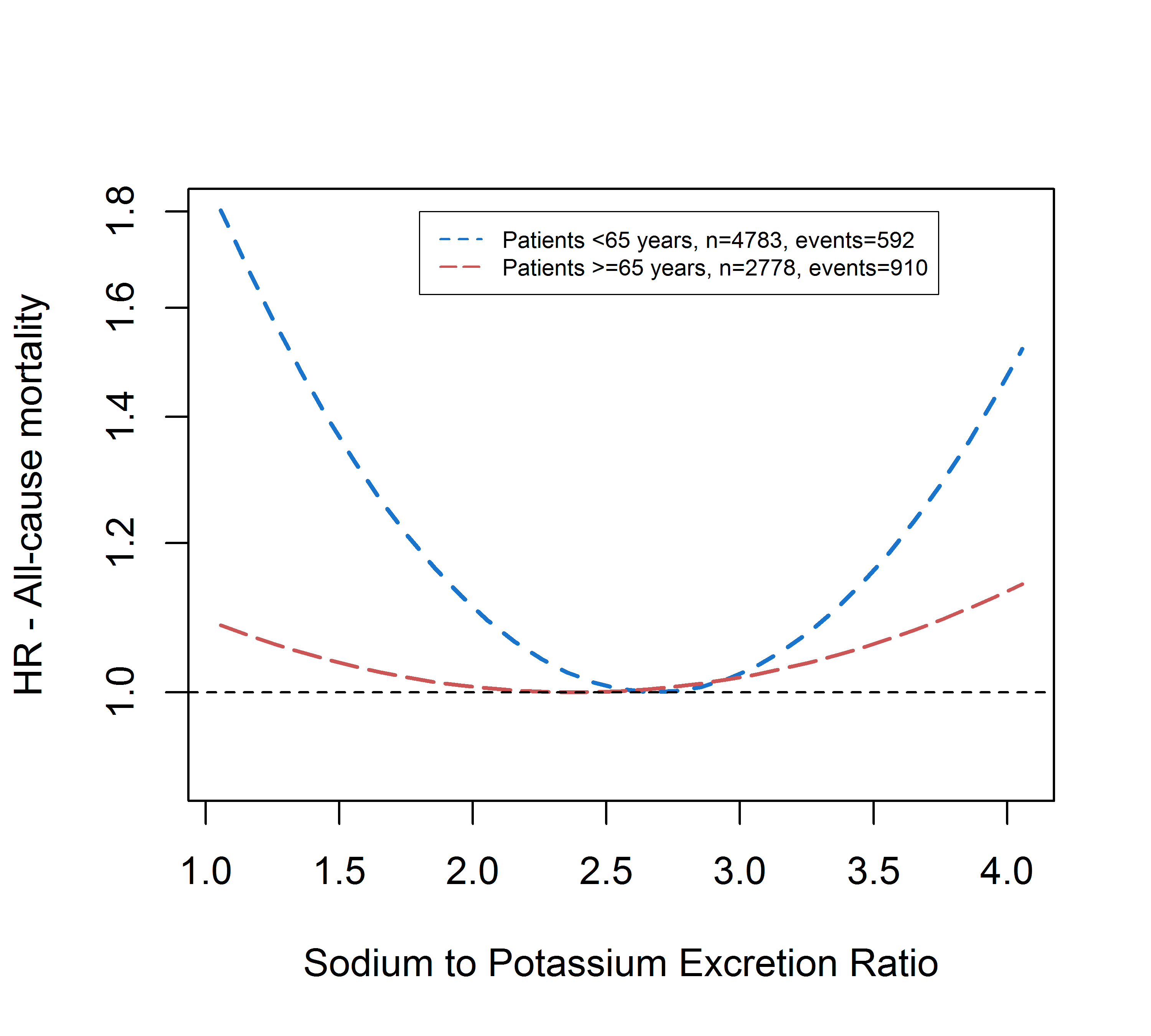

Supplement: S5 Fig — Adjusted hazard ratio for mortality by baseline sodium-to-potassium excretion ratio. Hazard ratios were plotted between the 1st and 99th percentile of the sodium-to-potassium excretion ratio. Plots were adjusted for age, sex, current smoking, BMI (kg/m2), presence of diabetes, eGFR, and non-high-density lipoprotein cholesterol. HR = Hazard ratio. (PNG) [file pone.0265429.s005.png]

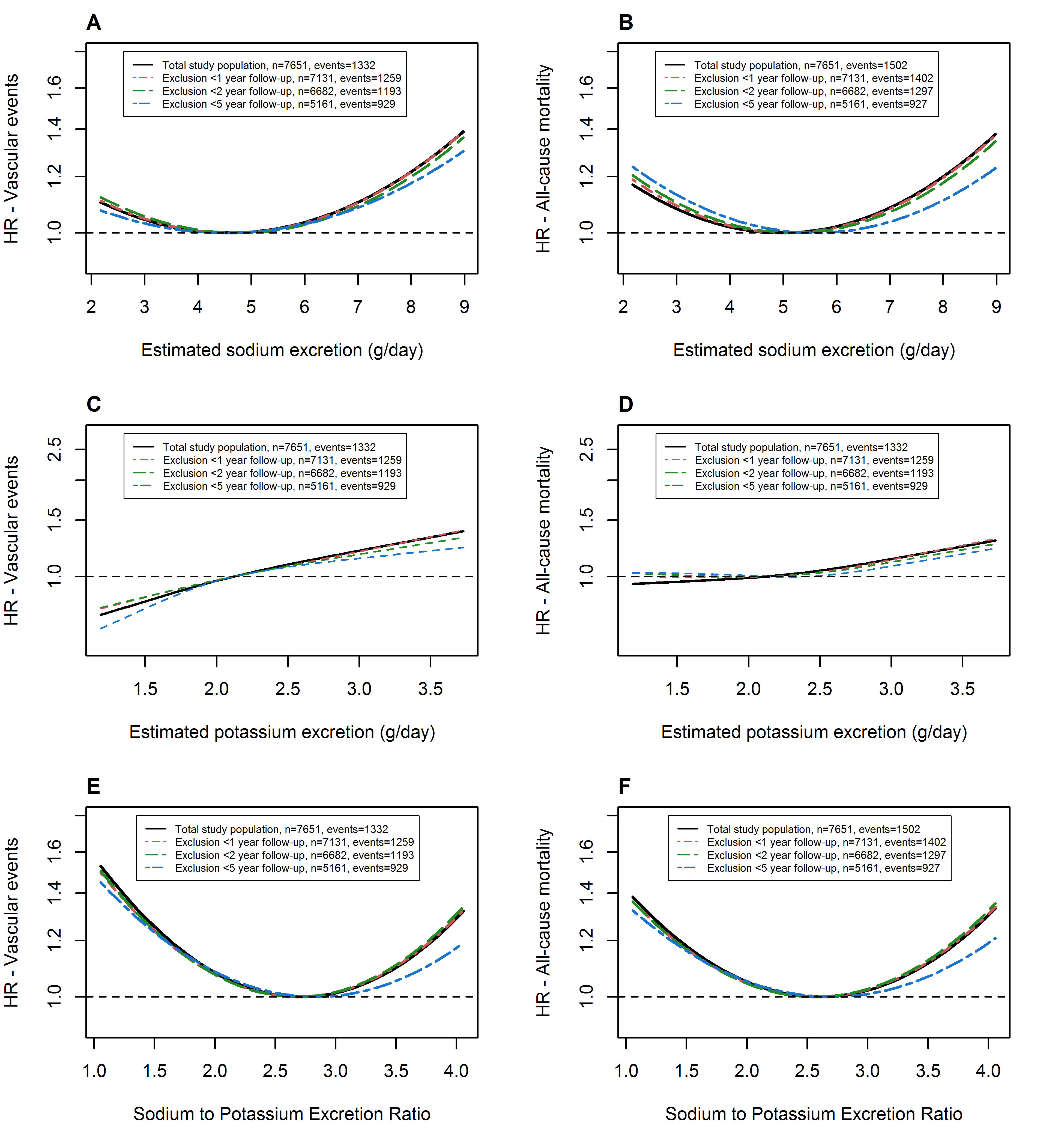

Supplement: S6 Fig — A-B. Change in estimated effect between estimated 24-hour sodium urinary excretion and vascular events (A) and mortality (B) after exclusion of patients who experienced events or died within 1 year (dashed red line), 2 years (dashed green line), and 5 years (dashed blue line) after inclusion. Black lines depict the main analysis. C-D. Change in estimated effect between 24-hour potassium urinary excretion and vascular events (C) and mortality (D) after exclusion of patients who experienced events or died within 1 year (dashed red line), 2 years (dashed green line), and 5 years (dashed blue line) after inclusion. E-F. Change in estimated effect between sodium-to-potassium excretion ratio and vascular events (E) and mortality (F) after exclusion of patients who experienced events or died within 1 year (dashed red line), 2 years (dashed green line), and 5 years (dashed blue line) after inclusion. HR = Hazard ratio. (PNG) [file pone.0265429.s006.png]

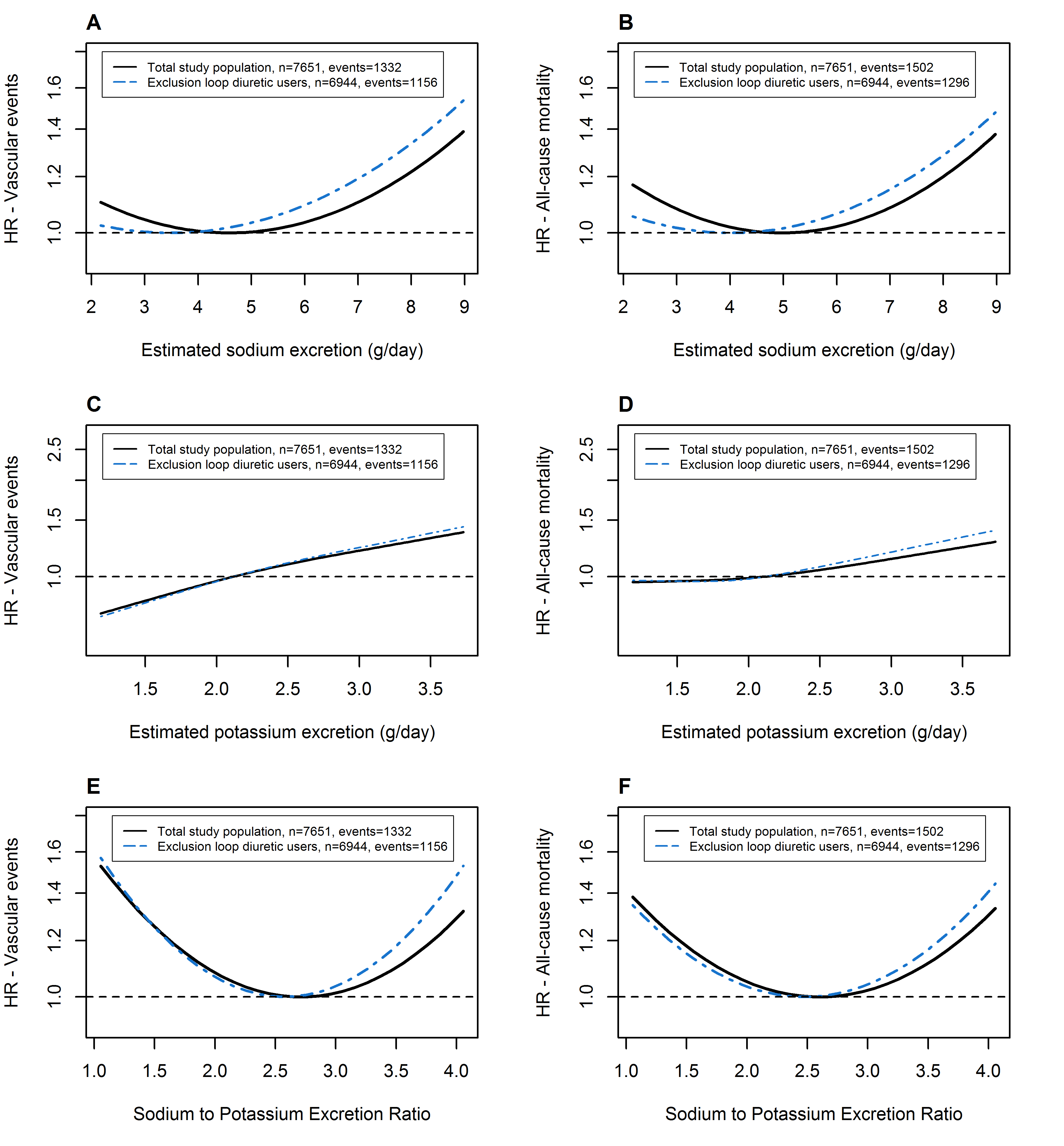

Supplement: S7 Fig — A-B. Change in estimated effect between estimated 24-hour sodium urinary excretion and vascular events (A) and mortality (B) after exclusion of patients who were treated with loop diuretics (dashed blue line). Black lines depict the main analysis. C-D. Change in estimated effect between 24-hour potassium urinary excretion and vascular events (C) and mortality (D) after exclusion of patients who were treated with loop diuretics (dashed blue line). E-F. Change in estimated effect between sodium-to-potassium excretion ratio and vascular events (E) and mortality (F) after exclusion of patients who were treated with loop diuretics (dashed blue line). HR = Hazard ratio. (PNG) [file pone.0265429.s007.png]
