# Supplementary material for: The relation between urinary sodium and potassium excretion and risk of cardiovascular events and mortality in patients with cardiovascular disease
Source: PLoS One. 2022 Mar 17;17(3):e0265429. doi: 10.1371/journal.pone.0265429 (PMC8929575; doi:10.1371/journal.pone.0265429)
Supplement: S1 Table — (DOCX) [file pone.0265429.s009.docx]

# S1 Table. Kawasaki formula used to predict 24-hour urinary sodium and potassium excretion from spot urine samples.

|  | Equation for estimating predicted 24-hour urine sodium or potassium excretion | Equation for estimating predicted 24-hour urine creatinine excretion (Pr24UCr mg/day) |
| --- | --- | --- |
| Sodium (mg/day) | 23 × (16.3 × XNa^0.5^), where XNA = [spot Na (mmol/  l)/spot creatinine (mg/dL) × 10] × Pr24UCr (mg/day) | Pr24UCr (mg/day) for men = (12.63 × age (year)) + (15.12 ×  weight (kg)) + (7.39 × height (cm))− 79.9  Pr24UCr (mg/day) for women = (− 4.72 × age (year)) +  (8.58 × weight (kg)) + (5.09 × height (cm))− 74.5 |
| Potassium (mg/day) | 39 × (7.2 × XK^0.5^), where XK = [spot K (mmol/  L)/spot creatinine (mg/dL) × 10] × Pr24UCr (mg/day) | Pr24UCr (mg/day) for men = (12.63 × age (year)) + (15.12 ×  weight (kg)) + (7.39 × height (cm))− 79.9  Pr24UCr (mg/day) for women = (− 4.72 × age (year)) +  (8.58 × weight (kg)) + (5.09 × height (cm))− 74.5 |
