# Supplementary material for: The relation between urinary sodium and potassium excretion and risk of cardiovascular events and mortality in patients with cardiovascular disease
Source: PLoS One. 2022 Mar 17;17(3):e0265429. doi: 10.1371/journal.pone.0265429 (PMC8929575; doi:10.1371/journal.pone.0265429)
Supplement: S2 Table — (DOCX) [file pone.0265429.s010.docx]

**S2 Table. Definitions of vascular outcomes.**

| **Outcome** | **Definition** |
| --- | --- |
| Myocardial infarction | (Non-)fatal myocardial infarction defined by ≥2 of the following:  -Acute chest pain for at least 20 min -ST-elevation >1 mm in two adjacent leads or a left bundle branch block (LBBB) on ECG  -Elevated troponin or elevated CK ≥2 times the normal value of CK and a MB-fraction >5% of the total CK;  Or;  - Coronary artery bypass graft (CABG) or percutaneous coronary intervention (PCI)  -Sudden death (unexpected cardiac death occurring within 1 hour after onset of symptoms, or within 24 hours given convincing circumstantial evidence). |
| Stroke | (Non-) fatal ischemic or hemorrhagic stroke: Relevant clinical features for at least 24 hours causing an increase in impairment of at least one grade of the modified Rankin scale, with/without a new infarction or hemorrhage on CT or MRI. |
| Vascular mortality | Death from myocardial infarction, stroke, heart failure, or rupture of abdominal aortic aneurysm; vascular death from other causes; or sudden death (unexpected cardiac death occurring within 1 hour after onset of symptoms, or within 24 hours given convincing circumstantial evidence)). |
| Major Adverse Cardiovascular Events (MACE) | Composite of the above mentioned outcomes |
| All-cause mortality | All deaths during follow-up, irrespective of the cause of death. |
