# Supplementary material for: The relation between urinary sodium and potassium excretion and risk of cardiovascular events and mortality in patients with cardiovascular disease
Source: PLoS One. 2022 Mar 17;17(3):e0265429. doi: 10.1371/journal.pone.0265429 (PMC8929575; doi:10.1371/journal.pone.0265429)
Supplement: S3 Table — (DOCX) [file pone.0265429.s011.docx]

**S3 Table. Baseline characteristics of all participants, according to estimated 24 hour potassium excretion.**

|  |  | **Estimated urinary potassium excretion, g/d; quintiles** | | | | |
| --- | --- | --- | --- | --- | --- | --- |
|  | **Overall** | **Q1** | **Q2** | **Q3** | **Q4** | **Q5** |
| Range quintiles (g/day) |  | **[0.72-1.76]** | **[1.77-2.01]** | **[2.02-2.25]** | **[2.26-2.57]** | **[2.58-7.09]** |
| Mean Potassium (g/day) | 2.2 ± 0.5 | 1.5 ± 0.2 | 1.9 ± 0.1 | 2.1 ± 0.1 | 2.4 ± 0.1 | 3.0 ± 0.4 |
|  | n = 7561 | n = 1513 | n = 1512 | n = 1512 | n = 1512 | n = 1512 |
| Male sex | 5574 (74%) | 784 (52%) | 1058 (70%) | 1162 (77%) | 1257 (83%) | 1313 (87%) |
| Age (years) | 60 (10%) | 58 ± 11 | 60 ± 10 | 61 ± 10 | 61 ± 10 | 61 ± 10 |
| Current smoker | 2396 (32%) | 552 (36%) | 502 (33%) | 441 (29%) | 472 (31%) | 429 (28%) |
| *Physical examination* |  |  |  |  |  |  |
| Body mass index (kg/m2) | 26.8 ± 4.0 | 26.0 ± 3.9 | 26.5 ± 4.0 | 26.9 ± 3.9 | 27.2 ± 3.9 | 27.5 ± 4.2 |
| Systolic blood pressure (mmHg) | 140 ± 21 | 138 ± 21 | 140 ± 21 | 140 ± 20 | 141 ± 21 | 141 ± 21 |
| Diastolic blood pressure (mmHg) | 81 ± 11 | 80± 11 | 81 ± 11 | 81 ± 11 | 81 ± 11 | 82 ± 12 |
| *History of vascular disease* |  |  |  |  |  |  |
| Diabetes mellitus | 1327 (18%) | 224 (15%) | 247 (16%) | 261 (17%) | 279 (18%) | 316 (21%) |
| Coronary artery disease | 4576 (61%) | 813 (54%) | 892 (59%) | 933 (62%) | 975 (64%) | 963 (64%) |
| Peripheral artery disease | 1408 (19%) | 307 (20%) | 296 (20%) | 250 (17%) | 293 (19%) | 262 (17%) |
| Cerebrovascular disease | 2247 (30%) | 517 (34%) | 473 (31%) | 451 (30%) | 388 (26%) | 418 (28%) |
| Abdominal aortic aneurysm | 650 (9%) | 109 (7%) | 125 (8%) | 116 (8%) | 144 (10%) | 156 (10%) |
| *Laboratory values* |  |  |  |  |  |  |
| Sodium excretion (g/day) | 4.9 ± 1.4 | 4.0 ± 1.1 | 4.6 ± 1.1 | 4.9 ± 1.2 | 5.2 ± 1.3 | 5.9 ± 1.6 |
| Total cholesterol (mmol/L) | 4.9 ± 1.2 | 5.0 ± 1.3 | 4.8 ± 1.2 | 4.8 ± 1.2 | 4.8 ± 1.1 | 4.8 ± 1.2 |
| HDL-cholesterol (mmol/L) | 1.2 ± 0.4 | 1.3 ± 0.4 | 1.2 ± 0.4 | 1.2 ± 0.4 | 1.2 ± 0.4 | 1.2 ± 0.4 |
| LDL-cholesterol (mmol/L) | 2.9 ± 1.1 | 3.0 ± 1.1 | 2.8 ± 1.1 | 2.9 ± 1.0 | 2.9 ± 1.0 | 2.8 ± 1.1 |
| Triglycerides (mmol/L) | 1.4 (1.0 - 2.0) | 1.4 (1.0 - 1.9) | 1.4 (1.0 - 2.0) | 1.4 (1.0 - 2.0) | 1.4 (1.0 - 2.0) | 1.5 (1.0 - 2.1) |
| Estimated GFR (ml/min/1.73m2) | 76 ± 18 | 78 ± 17 | 77 ± 17 | 75 ± 18 | 76 ± 18 | 76 ± 19 |
| CRP (mg/L) | 2.1 (1.0 - 4.4) | 2.0 (0.9 - 4.4) | 2.0 (1.0 - 4.4) | 2.1 (1.0 - 4.5) | 2.2 (1.0 - 4.4) | 2.0 (0.9 - 4.4) |
| *Medication use* |  |  |  |  |  |  |
| Lipid lowering | 5091 (67%) | 978 (65%) | 1006 (67%) | 1061 (70%) | 1009 (67%) | 1037 (69%) |
| Platelet inhibitor | 5762 (76%) | 1107 (73%) | 1147 (76%) | 1180 (78%) | 1161 (77%) | 1167 (77%) |
| Antihypertensives | 5599 (74%) | 1056 (70%) | 1110 (73%) | 1159 (77%) | 1127 (75%) | 1147 (76%) |
| Diuretics | 1574 (21%) | 324 (21%) | 303 (20%) | 341 (23%) | 288 (19%) | 318 (21%) |
| Loop diuretics | 617 (8%) | 115 (8%) | 118 (8%) | 130 (9%) | 120 (8%) | 134 (9%) |
| Thiazide diuretics | 874 (12%) | 189 (12%) | 179 (12%) | 191 (13%) | 151 (10%) | 164 (11%) |
| ACE-inhibitors | 2298 (30%) | 439 (29%) | 477 (32%) | 464 (31%) | 435 (29%) | 483 (32%) |
| Beta-blockers | 4023 (53%) | 776 (51%) | 770 (51%) | 851 (56%) | 811 (54%) | 815 (54%) |
| Calcium antagonists | 1568 (21%) | 260 (17%) | 320 (21%) | 315 (21%) | 342 (23%) | 331 (22%) |
| All data in n (%) or mean ± standard deviation (except for triglycerides and CRP: median with IQR). HDL, high-density lipoprotein; LDL, low-density lipoprotein; Hs-CRP, high-sensitivity C-reactive protein; BMI, body mass index; eGFR, estimated glomerular filtration rate (calculated with Chronic Kidney Disease Epidemiology Collaboration [CKD-EPI] formula). | | | | | | |
