# Supplementary material for: The relation between urinary sodium and potassium excretion and risk of cardiovascular events and mortality in patients with cardiovascular disease
Source: PLoS One. 2022 Mar 17;17(3):e0265429. doi: 10.1371/journal.pone.0265429 (PMC8929575; doi:10.1371/journal.pone.0265429)
Supplement: S4 Table — (DOCX) [file pone.0265429.s012.docx]

**S4 Table. P-values for interaction.**

|  | **Recurrent MACE** | | | **All-cause mortality** | | |
| --- | --- | --- | --- | --- | --- | --- |
| **Interaction variable:** | *24h Na excretion* | *24h K excretion* | *Na-to-K ratio* | *24h Na excretion* | *24h K excretion* | *Na-to-K ratio* |
| Sex | 0.93 | 0.35 | 0.54 | 0.52 | 0.89 | 0.95 |
| *quadratic term* | 0.86 |  | 0.59 | 0.51 |  | 0.88 |
| Age | 0.27 | 0.84 | 0.21 | 0.27 | 0.37 | 0.01* |
| *quadratic term* | 0.15 |  | 0.11 | 0.27 |  | 0.01* |
| Hypertension | 0.88 | 0.41 | 0.84 | 0.21 | 0.9 | 0.13 |
| *quadratic term* | 0.72 |  | 0.92 | 0.19 |  | 0.11 |
| Use of antihypertensive drugs | 0.11 | 0.53 | 0.11 | 0.35 | 0.41 | 0.18 |
| *quadratic term* | 0.06 |  | 0.11 | 0.19 |  | 0.12 |
| 24h K excretion | 0.82 |  |  | 0.42 |  |  |
| *quadratic term* | 0.27 |  |  | 0.11 |  |  |
| 24h Na excretion |  | 0.55 |  |  | 0.98 |  |
| *quadratic term* |  |  |  |  |  |  |

**significant interaction*
